# Post-translational Mechanisms Regulating NK Cell Activating Receptors and Their Ligands in Cancer: Potential Targets for Therapeutic Intervention

**DOI:** 10.3389/fimmu.2019.02557

**Published:** 2019-10-31

**Authors:** Rosa Molfetta, Alessandra Zingoni, Angela Santoni, Rossella Paolini

**Affiliations:** Department of Molecular Medicine, Sapienza University of Rome, Laboratory Affiliated to Istituto Pasteur Italia-Fondazione Cenci Bolognetti, Rome, Italy

**Keywords:** activating NK cell receptors, ligands for NK cell activating receptors, post-translational modifications, shedding, ubiquitin modification

## Abstract

Efficient clearance of transformed cells by Natural Killer (NK) cells is regulated by several activating receptors, including NKG2D, NCRs, and DNAM-1. Expression of these receptors as well as their specific “induced self” ligands is finely regulated during malignant transformation through the integration of different mechanisms acting on transcriptional, post-transcriptional, and post-translational levels. Among post-translational mechanisms, the release of activating ligands in the extracellular milieu through protease-mediated cleavage or by extracellular vesicle secretion represents some relevant cancer immune escape processes. Moreover, covalent modifications including ubiquitination and SUMOylation also contribute to negative regulation of NKG2D and DNAM-1 ligand surface expression resulting either in ligand intracellular retention and/or ligand degradation. All these mechanisms greatly impact on NK cell mediated recognition and killing of cancer cells and may be targeted to potentiate NK cell surveillance against tumors. Our mini review summarizes the main post-translational mechanisms regulating the expression of activating receptors and their ligands with particular emphasis on the contribution of ligand shedding and of ubiquitin and ubiquitin-like modifications in reducing target cell susceptibility to NK cell-mediated killing. Strategies aimed at inhibiting shedding of activating ligands and their modifications in order to preserve ligand expression on cancer cells will be also discussed.

## Introduction

Natural Killer (NK) cell activation is tuned by the integration of signals derived from inhibitory receptors for Major Histocompatibility Complex (MHC) class I molecules and from activating receptors that bind either non self-molecules associated to pathogens or self-molecules up-regulated in stress conditions including malignant transformation (1, 2).

Among activating receptors, Natural-Killer receptor group 2, member D (NKG2D), DNAX-associated molecule1 (DNAM-1), and the Natural Cytotoxicity Receptors (NCRs) play a pivotal role in NK cell-mediated tumor surveillance as revealed by an increased incidence of spontaneous malignancies or impaired tumor clearance in mice deficient for these receptors (3–7). In human, NCR expression may represent a prognostic biomarker in acute myeloid leukemia (AML) and solid tumors (8–10). Moreover, the engagement of the low affinity receptor for IgG (CD16) by means of natural or therapeutic monoclonal antibodies can also contribute to tumor clearance through antibody-dependent cellular cytotoxicity (ADCC) (11, 12).

### NK Cell Activating Receptors and Their Ligands on Tumor Cells

NKG2D is a C-type lectin receptor not exclusively expressed on NK cells but also found on NKT, CD8^+^αβ T cells, γδ T cells, and activated CD4^+^αβ T cells (13–15). In humans, NKG2D binds to the adaptor DNAX activating protein 10 (DAP10), responsible for signal propagation. In murine activated NK cells, a shorter NKG2D isoform can either associate with DAP10 or DAP12, an alternative signal transducing adaptor (16, 17).

Human NKG2D ligands (NKG2DLs) belong to two families of polymorphic molecules structurally related to MHC class I: the MHC class I related proteins (MIC)A/B which possess α1, α2, and α3 domains similar to MHC molecules and six UL16 binding proteins (ULBP1-6) characterized by α1 and α2 domains (15, 18, 19). MICA and MICB are generally transmembrane proteins, while ULBP proteins can be transmembrane (ULBP4 and 6) or GPI-linked (ULPB1-3 and 5) molecules. Murine NKG2DLs include Rae-1α-ε, MULT1, and H60a-c and are expressed either as transmembrane or GPI-linked molecules (18).

DNAM-1 belongs to the immunoglobulin receptor family and is expressed not only on NK cells but also on monocytes, T cells, and subsets of B cells (14, 20, 21). It binds to Nectin2/CD112 and PVR/CD155 both members of the Nectin/Nectin-like family of adhesion molecules (22–24), and it associates with the integrin LFA1 to transduce intracellular signals.

Natural cytotoxicity receptors comprise NKp46, NKp44, and NKp30 immunoglobulin-like receptors that are not exclusively expressed on NK cells but also on innate lymphoid cells (ILCs) of group 1 (ILC1) and a subset of ILC3, γδ T cells, and a population of cytotoxic T lymphocytes (25, 26). Only ortholog of NKp46 is expressed in mice (26).

NKp30 and NKp46 associate with the signal transducing adaptors CD3ζ and FcεRIγ while NKp44 mainly signals through the DAP12 adapter. Splicing variants of NKp44 and NKp30 endowed with inhibitory signal capability have been described and are associated with worst prognosis in cancer patients (9, 27).

NCRs interact with several ligands that are either pathogen-encoded or self-molecules and include cell surface and intracellular proteins that reach the surface in infected or transformed cells (28). However, the ligands expressed on tumor cells have not been fully identified yet.

Each NCR has the ability to recognize a specific configuration of heparan sulfate proteoglycans expressed in the context of tumor microenvironment, and this binding can modulate receptor function (28).

Ligands for NKp30 include B7-H6 belonging to the B7 family and only expressed on tumor cells, the intracellular protein HLA-B associated transcript 3 (BAT3), also known as BAG6, and galectin-3 (29–31). The first two ligands bind to and activate NKp30 while the released form of galectin-3 inhibits anti-tumor NKp30 function.

NKp44 interacts with the Proliferating Cell Nuclear Antigen (PCNA), which is aberrantly expressed on the surface of tumor cells. This binding preferentially engages an inhibitory isoform of NKp44 and negatively regulates NK cell functions (32). Interaction between NKp44 and a subset of HLA-DP molecules has been recently reported (33) demonstrating that HLA class II molecules may impact on NK cell activity. Of note, NKp44 can be triggered by specific tumor-derived soluble growth factors (34) and by Nidogen-1, an extracellular matrix protein (35). NKp46 recognize viral ligands including hemagglutinins as well as tumor ligands of still unknown identity.

Most of the above mentioned NK cell activating ligands, including NKG2DLs and B7-H6, are absent in normal cells but their expression is induced upon neoplastic transformation, thus rendering tumor cells more susceptible to NK cell-mediated killing (29, 36–41).

On the other hand, PVR and Nectin2 are expressed on healthy cells (21) but their amount is up-regulated on epithelial and hematological tumor cells promoting NK cell cytotoxicity (7, 42–44).

## Post-Translational Mechanisms Modulating Membrane Expression of NK Cell Activating Ligands on Tumor Cells

**Figure 1:**
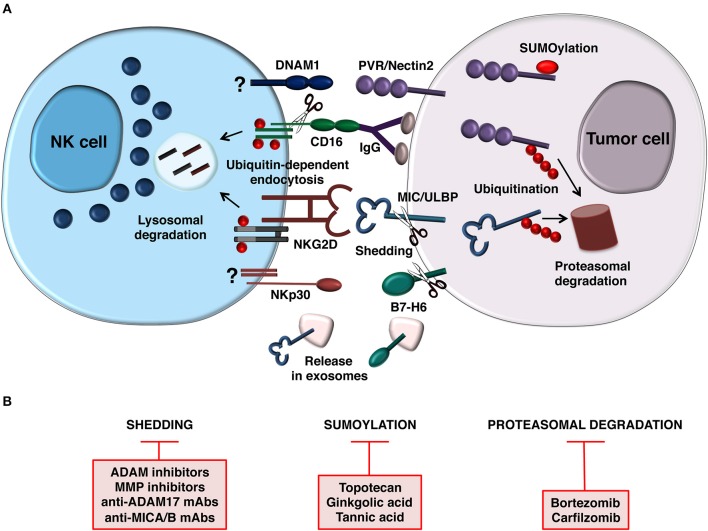
Post-translational mechanisms controlling NK cell-mediated recognition of tumor cells. **(A)** Model depicting how NCR (B7-H6), NKG2D, and DNAM1 ligand expression is prevented by post-translational mechanisms on target cell surface (right). Release of B7-H6 and NKG2D ligands on exosomes is also depicted. In addition to regulate ligand expression on tumor cell, ubiquitin modification also provides a signal for internalization and trafficking of NKG2D and CD16 on NK cells (left). CD16 is also downmodulated by metalloproteinase-mediated shedding. Mechanisms regulating DNAM-1 and NCR (NKp30) downmodulation are currently unknown. **(B)** Therapeutic strategies aimed to prevent post-translational mechanisms affecting activating ligand and receptor expression.

During malignant transformation different stressful stimuli are responsible for the induction of NK cell activating ligands at transcriptional and post-transcriptional levels and the molecular mechanisms implicated have been partially identified (18, 45). Moreover, increasing evidence demonstrate that post-translational mechanisms including the release of ligands for NK cell activating receptors as soluble forms as well as ligand modification by the Ubiquitin (Ub) or Ub-like pathways are used by tumor cells to dampen activating ligand surface expression in order to evade NK cell recognition (Figure 1A).

### Mechanisms Implicated in the Release of Ligands for NK Cell Activating Receptors

Most of the information regarding soluble ligands in cancer patients comes from studies performed on NKG2DLs. These molecules are present in the sera of patients affected by hematological or solid malignancies, and their level correlate with tumor stage and poor prognosis (46–53). More recently, B7-H6, BAG6, and PVR soluble forms have been found in the sera of patients affected by different type of tumors suggesting a relationship between soluble ligand expression and cancer progression (54–58).

Generation of soluble ligands relies on different mechanisms including alternative splicing, exosome secretion and proteolytic cleavage. Soluble PVR isoforms are generated by alternative splicing (59) and have an inhibitory effect on DNAM-1 mediated tumor immunity (54). In addition, alternative splicing gives rise to ULBP-4/5 secreted ligands that can impair NK cell target recognition *in vitro* (60, 61).

Exosomes represents nanovesicles derived from the endosomal compartment (62) and have been involved in the secretion of NKG2D and NKp30 ligands but not of DNAM-1 ligands (63). Differently from the proteolytic-mediated release, expression of activating ligands on the exosome surface should retain their biological activity by keeping the integral-molecule. A number of studies have shown that NKG2DLs from both MIC and ULBP families are expressed on the surface of exosome-like vesicles released from ovarian cancer (63), melanoma (64), and prostate cancer cells (65). Remarkably, NKG2DLs such as ULBP3 and ULBP1 (66) or the allelic variant MICA^*^008 (67, 68) that are glycosylphosphatidylinositol (GPI)-anchored proteins, are preferentially released via exosomes.

In regard to NKp30Ls, the nuclear protein BAG6 is secreted on exosomes and stimulates NK cell activity (69), whereas the cell surface ligand B7-H6 can be released in its soluble form associated to exosomes or through protease-mediated cleavage (57, 70, 71). Although several stress conditions can increase exosome secretion from cancer cells (72–75), it is still uncertain whether the release of NKG2DLs or B7-H6 through exosome-like vesicles could result in the diminution of their expression on the cell surface.

Concerning the shedding process, MICA, MICB, and ULBP2 are cut by metalloproteinases belonging to two distinct families, the matrix metalloproteinases (MMPs) and a disintegrin and metalloproteinases (ADAMs) (76–81), whereas the B7-H6 proteolytic cleavage occurs through a mechanism mainly dependent on ADAM enzymes (57). A recent study has shown that some ULBP4 isoforms are sensitive to the protease cleavage (82). Both MMPs and ADAMs proteases undergo modulation of their activity and expression in the course of neoplastic transformation (83, 84) and in response to cancer therapy (85–88). Disparate sensitivity to the proteases has been described for distinct NKG2DLs and/or allelic variants and isoforms. For instance, the generation of soluble MICA can be affected by polymorphisms as shown for the MICA^*^008 allele that is resistant to the protease-mediated cleavage. Moreover, the MICA-129 dimorphism, producing a valine to methionine swap at position 129, influenced the MICA cleavage process but the mechanism behind has to be defined (89, 90). In addition, proteolytic cleavage can be affected by fatty acylation and palmytolation that mediate MICA/B recruitment to membrane microdomains (78, 91).

Differently from the exosome-mediated release, the proteolytic cleavage of NKG2DLs and B7H6 has been associated to a reduction of cell surface ligands, thus its inhibition could be accomplished as a promising approach to keep the ligands on cancer cell surface and to promote anti-cancer immune response.

### Activating Ligand Modification by Ub and Ub-Like Pathways

Recent evidences reveal a role for ubiquitination and SUMOylation in the regulation of NK cell ligand expression on tumor cells.

Ubiquitination and SUMOylation are reversible modifications whereby Ub and small Ub-like modifier (SUMO), respectively, are covalently bound to a target protein through the action of enzymes frequently up-regulated during malignant transformation (92–95).

Once modified, proteins undergo different fate depending on the type of modification.

Proteins modified by poli-Ub chains are generally targeted to proteasomal degradation (95) whereas the addition of single Ub molecules to one or more lysine residues promote non-degradative fates including regulation of membrane protein endocytosis (96). SUMOylated substrates undergo conformational changes that in turn modify their interaction with other proteins or their enzymatic activity without inducing a degradative fate (94).

Little is currently known about the role of these modifications in the regulation of NK cell ligand expression during malignant transformation.

Ubiquitination of MICA/B has been demonstrated in Kaposi's sarcoma-associated herpesvirus infected cells: the viral E3 Ub ligase K5 induces modification of both NKG2DLs and their intracellular retention (97). Moreover, in healthy cells the murine ULBP-1 ortholog MULT-1 undergoes constitutive ubiquitination and lysosomal degradation (98, 99). Interestingly, stress conditions including UV radiation and heat shock prevent MULT-1 ubiquitination and increase its surface expression (98). Thus, these results support a negative role for the Ub pathway in the regulation of NKG2DL expression.

In tumor cells a direct implication of the Ub pathway has not been formally reported but several data demonstrate that surface expression of human NKG2DLs is regulated by a rapid protein turnover. In melanoma cells, an immature form of MICA accumulates in the endoplasmic reticulum and is targeted to degradation (100). MICB is internalized and retained intracellularly in several tumor cell lines (101), while in Multiple Myeloma (MM) cells the constitutive internalization of MICB is followed by its lysosomal degradation (102). Similarly, the GPI-linked ligand ULBP1 is continuously removed from plasma membrane and targeted to proteasomal degradation (103). Regarding DNAM-1 ligands, in hepatocellular carcinoma the activation of Unfolded Protein Response (UPR) inhibits PVR surface expression and promotes protein degradation (104). In line with this result, ubiquitination and SUMOylation negatively regulates surface expression of Nectin2 and PVR on tumor cells (105, 106). Ubiquitinated Nectin2 is retained in intracellular compartments but also targeted to proteasomal degradation (106) whereas SUMOylation of PVR promotes its intracellular retention without inducing protein degradation (105). Inhibition of Ub and SUMO pathways increases Nectin2 and PVR surface expression and renders tumor cells more sensitive to NK cell-mediated killing.

Although these findings are currently limited to NKG2D and DNAM-1 ligands, they provide novel insights into the mechanisms underlying activating ligand expression in diseased cells and reveal novel potential targets for therapeutic intervention.

## Ligand Induced Down-Modulation of NK Cell Activating Receptor Expression

Tumor progression also implies the inability of NK cells to kill tumor cells as consequence of ligand mediated down-regulation of activating receptors (Figure 1A).

However, receptor down-regulation may be affected by the presence of soluble or membrane-bound ligands as well as by their affinity and/or avidity (19).

A decreased in NCR expression levels was observed in NK cells derived from patients affected by myeloid leukemia and other tumors upon the interaction with their respective ligands (8, 107).

Reduced NKp30 surface expression has been also detected on NK cells derived from ovarian carcinoma and neuroblastoma patients as a result of chronic stimulation either with B7-H6-expressing tumor cells or soluble B7-H6 (55, 56, 108). Moreover, the presence of soluble BAG6 has been associated with a low transcriptional levels of different NKp30 isoforms (58, 109).

DNAM-1 engagement, by membrane-bound ligands but not their soluble counterpart, is also followed by receptor down-modulation and impairment of NK cell functions in patients affected by different tumors including MM, ovarian carcinoma and AML (44, 110, 111). However, the mechanisms underlying these effects are still undefined.

For other activating receptors including NKG2D and CD16, mechanisms of ligand-induced down-modulation have been elucidated.

NKG2D stimulation by ligands expressed on tumor cells as well as by soluble ligands promotes receptor endocytosis and the decrease of NKG2D-dependent functions (46, 76, 112–115). In regard to released ligands, those associated to exosomes show a higher avidity and a more efficient ability to induce receptor down-regulation compared to shed ligands (66, 67).

In humans, internalization of ligand-engaged NKG2D receptors requires DAP10 ubiquitination and is followed by lysosomal degradation (116). However, MICA is more efficient than ULBP2 in promoting receptor ubiquitination (114).

Ub modification has been also implicated in the down-modulation of CD16 in response to antibody-coated tumor cells (117–119). Indeed, CD16 clearance from NK cell surface is mainly induced by Ub-dependent endocytosis of aggregated receptors followed by degradation of CD16ζ subunit and the associated kinases (114, 117, 120). However, CD16 down-regulation can also occur as a consequence of metalloproteinase-induced receptor shedding (121–124). Regardless, NK cell-mediated ADCC, natural cytotoxicity, and the efficacy of antibody-based therapies resulted impaired (118, 119, 125).

Altogether these results demonstrate that activating receptor expression is modulated in tumor microenvironment by the interaction with ligand-expressing cells, thus impairing NK cell ability to counteract tumor development.

## Targeting Post-Translational Mechanisms Regulating NK Cell-Mediated Recognition and Killing of Cancer Cells

All these post-translational mechanisms represent potential targets for therapeutic intervention (Figure 1B). Ligand shedding blocking can be achieved by the usage of inhibitors of MMPs and ADAMs enzymes (57, 77, 126). Since ADAM10 and ADAM17 sheddases play a prominent role in B7-H6 (57) and NKG2DL cleavage (77, 78, 80, 127), the selective targeting of such enzymes might be promising for anticancer therapy. Recently, by performing an *in vitro* drug screen using an FDA-approved drug library, lomofungin was found to strongly decrease ADAM17 activity in hepatocellular carcinoma leading to the impairment of MICA shedding and has been proposed as new drug candidate for immunotherapy in liver cancer (128). Most of the compounds able to inhibit ADAM catalytic activity are hydroxamate-based and are either selective for ADAM17 or inhibitors of both ADAM10 and ADAM17 (129). Of interest, the synthesis of new selective ADAM10 inhibitors able to impair NKG2DL shedding in Hodgkin's lymphoma cell models has been reported (130). ADAM10 and ADAM17 are expressed at high levels on the surface of glioblastoma-initiating cells thus contributing to an immunosuppressive phenotype through the ULBP2 cleavage. Specific inhibition of these enzymes preserved cell surface ULBP2 leading to increased glioblastoma cell recognition and killing by NK cells (131). Remarkably, *in vivo* experiments using athymic nu-/nu- mice implanted with subcutaneous HeLa tumors demonstrated that systemic MMPi treatment resulted in the reduction of MICA serum levels and a concomitant augmentation of MICA expression on cancer cells reinforcing the immune cell therapy mediated by cytokine-induced killer cells (132). Of interest, adoptively transferred NK cells displaying high levels of surface NKG2D determined the clearance of soluble MICA in neuroblastoma patients by preserving NK cell cytotoxicity via non-occupied NKG2D (133).

Another appealing strategy to specifically inhibit MICA/B proteolytic cleavage concerns the generation of antibodies targeting the MIC protein domain involved in the proteolytic cleavage (134). Interestingly the usage of these antibodies limited MICA/B shedding in human cancer cells and repressed cancer cell growth in *in vivo* models (134). More recently, the glycosylation-engineered epitope mapping (GEM) method allowed to the identification of a number of epitopes relevant for MICA/B shedding inhibition (135).

In general, such antibodies as well as metalloproteinase inhibitors could be used in combination with other therapies aimed at the enhancement of ligand expression on the surface of cancer cells including DNA damaging agents (127), radiations (87), and chemotherapeutic drugs (132). Our group has shown that the combined use of metalloproteinase inhibitors and genotoxic drugs enhanced NK cell-mediated killing of multiple myeloma cells by preserving MIC molecules on the cell surface (127). To date, ADAMs inhibitors have been largely unsuccessful in clinical trials, but they remain a viable and desirable therapeutic target based on preclinical studies.

Strategies aimed at inhibiting ADAM17-mediated CD16 cleavage from the surface of NK cells could be also promising. Beyond the usage of inhibitors, recent advances in generating function-blocking antibodies of ADAM17 are emerging. The monoclonal antibody MEDI3622 has been shown to block CD16A cleavage from activated human NK cells allowing to an increased IFNγ production in the course of ADCC (136).

Proteasome inhibitors can change the fate of ubiquitinated ligands. Bortezomib (Velcade) and Carfilzomib (Kyprolis) have been already used as chemotherapeutic drugs for relapsed MM patients (137–139) and for the treatment of mantle cell lymphoma (140).

In line with our findings (106), previous reports demonstrated that low doses of bortezomib increase NK cell activating ligand, including Nectin2 (141–143). Whether those drugs can directly affect ligand expression stabilizing ubiquitinated Nectin2 and/or SUMOylated PVR is currently unknown.

Regarding the SUMO pathway, the FDA-approved drug Topotecan has been shown to affect SUMOylation in glioblastoma multiforme (144). Moreover, natural compounds including ginkgolic acid and tannic acid (145, 146) have been found to possess anti-cancer activities by targeting the SUMO pathway (147).

All of these compounds hold great promise to be developed into novel and efficient anti-cancer drugs.

## Conclusion and Therapeutic Perspectives

On tumor cells, several activating ligands are subjected to protease-mediated cleavage with a consequent dramatic reduction of their surface expression. A similar effect is also achieved upon ubiquitination or SUMOylation of NKG2D and DNAM-1 ligands, which are retained intracellularly and/or degraded.

On NK cells, the Ub pathway may also contribute to down-regulate the surface expression of activating receptors.

In conclusion, all these post-translational mechanisms act to reduce NK cell-mediated surveillance against tumors and represent potential targets for therapeutic intervention.

Several inhibitors have been developed and their use in combination with conventional therapies represent a useful tool to potentiate NK-cell mediated recognition and killing of tumor cells.

## Author Contributions

RM, AZ, AS, and RP participated in the conception, writing, and elaboration of the final version of the manuscript.

### Conflict of Interest

The authors declare that the research was conducted in the absence of any commercial or financial relationships that could be construed as a potential conflict of interest.

## References

[1] Long EO Kim HS Liu D Peterson ME Rajagopalan S. Controlling natural killer cell responses: integration of signals for activation and inhibition. Ann Rev Immunol. (2013) 31:227–58. 10.1146/annurev-immunol-020711-075005 23516982 PMC3868343

[2] Morvan MG Lanier LL. NK cells and cancer: you can teach innate cells new tricks. Nat Rev Cancer. (2016) 16:7–19. 10.1038/nrc.2015.5 26694935

[3] Guerra N Tan YX Joncker NT Choy A Gallardo F Xiong N. NKG2D-deficient mice are defective in tumor surveillance in models of spontaneous malignancy. Immunity. (2008) 28:571–80. 10.1016/j.immuni.2008.02.016 18394936 PMC3528789

[4] Iguchi-Manaka A Kai H Yamashita Y Shibata K Tahara-Hanaoka S Honda S. Accelerated tumor growth in mice deficient in DNAM-1 receptor. J Exp Med. (2008) 205:2959–64. 10.1084/jem.20081611 19029379 PMC2605241

[5] Gilfillan S Chan CJ Cella M Haynes NM Rapaport AS Boles KS. DNAM-1 promotes activation of cytotoxic lymphocytes by nonprofessional antigen-presenting cells and tumors. J Exp Med. (2008) 205:2965–73. 10.1084/jem.20081752 19029380 PMC2605240

[6] Halfteck GG Elboim M Gur C Achdout H Ghadially H Mandelboim O. Enhanced *in vivo* growth of lymphoma tumors in the absence of the NK-activating receptor NKp46/NCR1. J Immunol. (2009) 182:2221–30. 10.4049/jimmunol.0801878 19201876

[7] Lakshmikanth T Burke S Ali TH Kimpfler S Ursini F Ruggeri L. NCRs and DNAM-1 mediate NK cell recognition and lysis of human and mouse melanoma cell lines *in vitro* and *in vivo*. J Clin Invest. (2009) 119:1251–63. 10.1172/JCI36022 19349689 PMC2673866

[8] Fauriat C Just-Landi S Mallet F Arnoulet C Sainty D Olive D. Deficient expression of NCR in NK cells from acute myeloid leukemia: evolution during leukemia treatment and impact of leukemia cells in NCRdull phenotype induction. Blood. (2007) 109:323–30. 10.1182/blood-2005-08-027979 16940427

[9] Delahaye NF Rusakiewicz S Martins I Menard C Roux S Lyonnet L. Alternatively spliced NKp30 isoforms affect the prognosis of gastrointestinal stromal tumors. Nat Med. (2011) 17:700–7. 10.1038/nm.2366 21552268

[10] Chretien AS Fauriat C Orlanducci F Rey J Borg GB Gautherot E. NKp30 expression is a prognostic immune biomarker for stratification of patients with intermediate-risk acute myeloid leukemia. Oncotarget. (2017) 8:49548–63. 10.18632/oncotarget.17747 28548938 PMC5564787

[11] Mentlik James A Cohen AD Campbell KS. Combination immune therapies to enhance anti-tumor responses by NK cells. Front Immunol. (2013) 4:481. 10.3389/fimmu.2013.00481 24391651 PMC3870292

[12] Battella S Cox MC Santoni A Palmieri G. Natural killer (NK) cells and anti-tumor therapeutic mAb: unexplored interactions. J Leukocyte Biol. (2016) 99:87–96. 10.1189/jlb.5VMR0415-141R 26136506

[13] Ullrich E Koch J Cerwenka A Steinle A. New prospects on the NKG2D/NKG2DL system for oncology. Oncoimmunology. (2013) 2:e26097. 10.4161/onci.26097 24353908 PMC3862635

[14] Marcus A Gowen BG Thompson TW Iannello A Ardolino M Deng W. Recognition of tumors by the innate immune system and natural killer cells. Adv Immunol. (2014) 122:91–128. 10.1016/B978-0-12-800267-4.00003-1 24507156 PMC4228931

[15] Lanier LL. NKG2D receptor and its ligands in host defense. Cancer Immunol Res. (2015) 3:575–82. 10.1158/2326-6066.CIR-15-0098 26041808 PMC4457299

[16] Diefenbach A Tomasello E Lucas M Jamieson AM Hsia JK Vivier E. Selective associations with signaling proteins determine stimulatory versus costimulatory activity of NKG2D. Nat Immunol. (2002) 3:1142–9. 10.1038/ni858 12426565

[17] Gilfillan S Ho EL Cella M Yokoyama WM Colonna M. NKG2D recruits two distinct adapters to trigger NK cell activation and costimulation. Nat Immunol. (2002) 3:1150–5. 10.1038/ni857 12426564

[18] Raulet DH Gasser S Gowen BG Deng W Jung H. Regulation of ligands for the NKG2D activating receptor. Ann Rev Immunol. (2013) 31:413–41. 10.1146/annurev-immunol-032712-095951 23298206 PMC4244079

[19] Zingoni A Molfetta R Fionda C Soriani A Paolini R Cippitelli M NKG2D and its ligands: “one for all, all for one”. Front Immunol. (2018) 9:476 10.3389/fimmu.2018.00476 29662484 PMC5890157

[20] Shibuya A Campbell D Hannum C Yssel H Franz-Bacon K McClanahan T. DNAM-1, a novel adhesion molecule involved in the cytolytic function of T lymphocytes. Immunity. (1996) 4:573–81. 10.1016/S1074-7613(00)70060-4 8673704

[21] de Andrade LF Smyth MJ Martinet L. DNAM-1 control of natural killer cells functions through nectin and nectin-like proteins. Immunol Cell Biol. (2014) 92:237–44. 10.1038/icb.2013.95 24343663

[22] Bottino C Castriconi R Pende D Rivera P Nanni M Carnemolla B. Identification of PVR (CD155) and Nectin-2 (CD112) as cell surface ligands for the human DNAM-1 (CD226) activating molecule. J Exp Med. (2003) 198:557–67. 10.1084/jem.20030788 12913096 PMC2194180

[23] Tahara-Hanaoka S Shibuya K Onoda Y Zhang H Yamazaki S Miyamoto A. Functional characterization of DNAM-1 (CD226) interaction with its ligands PVR (CD155) and nectin-2 (PRR-2/CD112). Int Immunol. (2004) 16:533–8. 10.1093/intimm/dxh059 15039383

[24] Chan CJ Smyth MJ Martinet L. Molecular mechanisms of natural killer cell activation in response to cellular stress. Cell Death Different. (2014) 21:5–14. 10.1038/cdd.2013.26 23579243 PMC3857624

[25] Moretta A Bottino C Vitale M Pende D Cantoni C Mingari MC. Activating receptors and coreceptors involved in human natural killer cell-mediated cytolysis. Ann Rev Immunol. (2001) 19:197–223. 10.1146/annurev.immunol.19.1.197 11244035

[26] Barrow AD Martin CJ Colonna M. The natural cytotoxicity receptors in health and disease. Front Immunol. (2019) 10:909. 10.3389/fimmu.2019.00909 31134055 PMC6514059

[27] Campbell KS Yusa S Kikuchi-Maki A Catina TL NKp44 triggers NK cell activation through DAP12 association that is not influenced by a putative cytoplasmic inhibitory sequence. J Immunol. (2004) 172:899–906. 10.4049/jimmunol.172.2.899 14707061

[28] Pazina T Shemesh A Brusilovsky M Porgador A Campbell KS. Regulation of the functions of natural cytotoxicity receptors by interactions with diverse ligands and alterations in splice variant expression. Front Immunol. (2017) 8:369. 10.3389/fimmu.2017.00369 28424697 PMC5371597

[29] Brandt CS Baratin M Yi EC Kennedy J Gao Z Fox B. The B7 family member B7-H6 is a tumor cell ligand for the activating natural killer cell receptor NKp30 in humans. J Exp Med. (2009) 206:1495–503. 10.1084/jem.20090681 19528259 PMC2715080

[30] Pogge von Strandmann E Simhadri VR von Tresckow B Sasse S Reiners KS Hansen HP. Human leukocyte antigen-B-associated transcript 3 is released from tumor cells and engages the NKp30 receptor on natural killer cells. Immunity. (2007) 27:965–74. 10.1016/j.immuni.2007.10.010 18055229

[31] Wang W Guo H Geng J Zheng X Wei H Sun R. Tumor-released Galectin-3, a soluble inhibitory ligand of human NKp30, plays an important role in tumor escape from NK cell attack. J Biol Chem. (2014) 289:33311–9. 10.1074/jbc.M114.603464 25315772 PMC4246088

[32] Rosental B Brusilovsky M Hadad U Oz D Appel MY Afergan F. Proliferating cell nuclear antigen is a novel inhibitory ligand for the natural cytotoxicity receptor NKp44. J Immunol. (2011) 187:5693–702. 10.4049/jimmunol.1102267 22021614 PMC3269963

[33] Niehrs A Garcia-Beltran WF Norman PJ Watson GM Holzemer A Chapel A. A subset of HLA-DP molecules serve as ligands for the natural cytotoxicity receptor NKp44. Nat Immunol. (2019) 20:1129–37. 10.1038/s41590-019-0448-4 31358998 PMC8370669

[34] Barrow AD Edeling MA Trifonov V Luo J Goyal P Bohl B. Natural killer cells control tumor growth by sensing a growth factor. Cell. (2018) 172:534–48 e19. 10.1016/j.cell.2017.11.037 29275861 PMC6684025

[35] Gaggero S Bruschi M Petretto A Parodi M Del Zotto G Lavarello C. Nidogen-1 is a novel extracellular ligand for the NKp44 activating receptor. Oncoimmunology. (2018) 7:e1470730. 10.1080/2162402X.2018.1470730 30228939 PMC6140582

[36] Groh V Rhinehart R Secrist H Bauer S Grabstein KH Spies T. Broad tumor-associated expression and recognition by tumor-derived gamma delta T cells of MICA and MICB. Proc Natl Acad Sci USA. (1999) 96:6879–84. 10.1073/pnas.96.12.6879 10359807 PMC22010

[37] Pende D Cantoni C Rivera P Vitale M Castriconi R Marcenaro S. Role of NKG2D in tumor cell lysis mediated by human NK cells: cooperation with natural cytotoxicity receptors and capability of recognizing tumors of nonepithelial origin. Eur J Immunol. (2001) 31:1076–86. 10.1002/1521-4141(200104)31:4<1076::AID-IMMU1076>3.3.CO;2-P 11298332

[38] Jinushi M Takehara T Tatsumi T Kanto T Groh V Spies T. Expression and role of MICA and MICB in human hepatocellular carcinomas and their regulation by retinoic acid. Int J Cancer. (2003) 104:354–61. 10.1002/ijc.10966 12569559

[39] Salih HR Antropius H Gieseke F Lutz SZ Kanz L Rammensee HG. Functional expression and release of ligands for the activating immunoreceptor NKG2D in leukemia. Blood. (2003) 102:1389–96. 10.1182/blood-2003-01-0019 12714493

[40] Friese MA Platten M Lutz SZ Naumann U Aulwurm S Bischof F. MICA/NKG2D-mediated immunogene therapy of experimental gliomas. Cancer Res. (2003) 63:8996–9006. 14695218

[41] Textor S Bossler F Henrich KO Gartlgruber M Pollmann J Fiegler N. The proto-oncogene Myc drives expression of the NK cell-activating NKp30 ligand B7-H6 in tumor cells. Oncoimmunology. (2016) 5:e1116674. 10.1080/2162402X.2015.1116674 27622013 PMC5007025

[42] Pende D Spaggiari GM Marcenaro S Martini S Rivera P Capobianco A. Analysis of the receptor-ligand interactions in the natural killer-mediated lysis of freshly isolated myeloid or lymphoblastic leukemias: evidence for the involvement of the Poliovirus receptor (CD155) and Nectin-2 (CD112). Blood. (2005) 105:2066–73. 10.1182/blood-2004-09-3548 15536144

[43] Carlsten M Bjorkstrom NK Norell H Bryceson Y van Hall T Baumann BC. DNAX accessory molecule-1 mediated recognition of freshly isolated ovarian carcinoma by resting natural killer cells. Cancer Res. (2007) 67:1317–25. 10.1158/0008-5472.CAN-06-2264 17283169

[44] El-Sherbiny YM Meade JL Holmes TD McGonagle D Mackie SL Morgan AW. The requirement for DNAM-1, NKG2D, and NKp46 in the natural killer cell-mediated killing of myeloma cells. Cancer Res. (2007) 67:8444–9. 10.1158/0008-5472.CAN-06-4230 17875681

[45] Cerboni C Fionda C Soriani A Zingoni A Doria M Cippitelli M. The DNA damage response: a common pathway in the regulation of NKG2D and DNAM-1 ligand expression in normal, infected, and cancer cells. Front Immunol. (2014) 4:508. 10.3389/fimmu.2013.00508 24432022 PMC3882864

[46] Doubrovina ES Doubrovin MM Vider E Sisson RB O'Reilly RJ Dupont B. Evasion from NK cell immunity by MHC class I chain-related molecules expressing colon adenocarcinoma. J Immunol. (2003) 171:6891–9. 10.4049/jimmunol.171.12.6891 14662896

[47] Rebmann V Schutt P Brandhorst D Opalka B Moritz T Nowrousian MR. Soluble MICA as an independent prognostic factor for the overall survival and progression-free survival of multiple myeloma patients. Clin Immunol. (2007) 123:114–20. 10.1016/j.clim.2006.11.007 17218152

[48] Jinushi M Vanneman M Munshi NC Tai YT Prabhala RH Ritz J. MHC class I chain-related protein A antibodies and shedding are associated with the progression of multiple myeloma. Proc Natl Acad Sci USA. (2008) 105:1285–90. 10.1073/pnas.0711293105 18202175 PMC2234130

[49] Wu JD Higgins LM Steinle A Cosman D Haugk K Plymate SR. Prevalent expression of the immunostimulatory MHC class I chain-related molecule is counteracted by shedding in prostate cancer. J Clin Invest. (2004) 114:560–8. 10.1172/JCI200422206 15314693 PMC503776

[50] Holdenrieder S Stieber P Peterfi A Nagel D Steinle A Salih HR. Soluble MICB in malignant diseases: analysis of diagnostic significance and correlation with soluble MICA. Cancer Immunol Immunother. (2006) 55:1584–9. 10.1007/s00262-006-0167-1 16636811 PMC11030555

[51] Holdenrieder S Stieber P Peterfi A Nagel D Steinle A Salih HR. Soluble MICA in malignant diseases. Int J Cancer. (2006) 118:684–7. 10.1002/ijc.21382 16094621

[52] Baragano Raneros A Suarez-Alvarez B Lopez-Larrea C. Secretory pathways generating immunosuppressive NKG2D ligands: New targets for therapeutic intervention. Oncoimmunology. (2014) 3:e28497. 10.4161/onci.28497 25050215 PMC4063154

[53] Paschen A Sucker A Hill B Moll I Zapatka M Nguyen XD. Differential clinical significance of individual NKG2D ligands in melanoma: soluble ULBP2 as an indicator of poor prognosis superior to S100B. Clin Cancer Res. (2009) 15:5208–15. 10.1158/1078-0432.CCR-09-0886 19671853

[54] Iguchi-Manaka A Okumura G Kojima H Cho Y Hirochika R Bando H. Increased soluble CD155 in the serum of cancer patients. PLoS ONE. (2016) 11:e0152982. 10.1371/journal.pone.0152982 27049654 PMC4822883

[55] Semeraro M Rusakiewicz S Minard-Colin V Delahaye NF Enot D Vely F. Clinical impact of the NKp30/B7-H6 axis in high-risk neuroblastoma patients. Sci Trans Med. (2015) 7:283ra55. 10.1126/scitranslmed.aaa2327 25877893

[56] Pesce S Tabellini G Cantoni C Patrizi O Coltrini D Rampinelli F. B7-H6-mediated downregulation of NKp30 in NK cells contributes to ovarian carcinoma immune escape. Oncoimmunology. (2015) 4:e1001224. 10.1080/2162402X.2014.1001224 26137398 PMC4485754

[57] Schlecker E Fiegler N Arnold A Altevogt P Rose-John S Moldenhauer G. Metalloprotease-mediated tumor cell shedding of B7-H6, the ligand of the natural killer cell-activating receptor NKp30. Cancer Res. (2014) 74:3429–40. 10.1158/0008-5472.CAN-13-3017 24780758

[58] Rusakiewicz S Perier A Semeraro M Pitt JM Pogge von Strandmann E Reiners KS. NKp30 isoforms and NKp30 ligands are predictive biomarkers of response to imatinib mesylate in metastatic GIST patients. Oncoimmunology. (2017) 6:e1137418. 10.1080/2162402X.2015.1137418 28197361 PMC5283614

[59] Baury B Masson D McDermott BM Jr Jarry A Blottiere HM Blanchardie P. Identification of secreted CD155 isoforms. Biochem Biophys Res Commun. (2003) 309:175–82. 10.1016/S0006-291X(03)01560-2 12943679

[60] Bacon L Eagle RA Meyer M Easom N Young NT Trowsdale J. Two human ULBP/RAET1 molecules with transmembrane regions are ligands for NKG2D. J Immunol. (2004) 173:1078–84. 10.4049/jimmunol.173.2.1078 15240696

[61] Cao W Xi X Hao Z Li W Kong Y Cui L. RAET1E2, a soluble isoform of the UL16-binding protein RAET1E produced by tumor cells, inhibits NKG2D-mediated NK cytotoxicity. J Biol Chem. (2007) 282:18922–8. 10.1074/jbc.M702504200 17470428

[62] Mathieu M Martin-Jaular L Lavieu G Thery C. Specificities of secretion and uptake of exosomes and other extracellular vesicles for cell-to-cell communication. Nat Cell Biol. (2019) 21:9–17. 10.1038/s41556-018-0250-9 30602770

[63] Labani-Motlagh A Israelsson P Ottander U Lundin E Nagaev I Nagaeva O. Differential expression of ligands for NKG2D and DNAM-1 receptors by epithelial ovarian cancer-derived exosomes and its influence on NK cell cytotoxicity. Tumour Biol. (2016) 37:5455–66. 10.1007/s13277-015-4313-2 26563374

[64] Lopez-Cobo S Campos-Silva C Moyano A Oliveira-Rodriguez M Paschen A Yanez-Mo M. Immunoassays for scarce tumour-antigens in exosomes: detection of the human NKG2D-Ligand, MICA, in tetraspanin-containing nanovesicles from melanoma. J Nanobiotechnol. (2018) 16:47. 10.1186/s12951-018-0372-z 29720199 PMC5932892

[65] Lundholm M Schroder M Nagaeva O Baranov V Widmark A Mincheva-Nilsson L. Prostate tumor-derived exosomes down-regulate NKG2D expression on natural killer cells and CD8+ T cells: mechanism of immune evasion. PLoS ONE. (2014) 9:e108925. 10.1371/journal.pone.0108925 25268476 PMC4182531

[66] Fernandez-Messina L Ashiru O Boutet P Aguera-Gonzalez S Skepper JN Reyburn HT. Differential mechanisms of shedding of the glycosylphosphatidylinositol (GPI)-anchored NKG2D ligands. J Biol Chem. (2010) 285:8543–51. 10.1074/jbc.M109.045906 20080967 PMC2838276

[67] Ashiru O Boutet P Fernandez-Messina L Aguera-Gonzalez S Skepper JN Vales-Gomez M. Natural killer cell cytotoxicity is suppressed by exposure to the human NKG2D ligand MICA^*^008 that is shed by tumor cells in exosomes. Cancer Res. (2010) 70:481–9. 10.1158/0008-5472.CAN-09-1688 20068167 PMC2817492

[68] Ashiru O Lopez-Cobo S Fernandez-Messina L Pontes-Quero S Pandolfi R Reyburn HT. A GPI anchor explains the unique biological features of the common NKG2D-ligand allele MICA^*^008. Biochem J. (2013) 454:295–302. 10.1042/BJ20130194 23772752

[69] Dassler-Plenker J Reiners KS van den Boorn JG Hansen HP Putschli B Barnert S. RIG-I activation induces the release of extracellular vesicles with antitumor activity. Oncoimmunology. (2016) 5:e1219827. 10.1080/2162402X.2016.1219827 27853642 PMC5087302

[70] Gutierrez-Franco J Hernandez-Gutierrez R Bueno-Topete MR Haramati J Navarro-Hernandez RE Escarra-Senmarti M. Characterization of B7H6, an endogenous ligand for the NK cell activating receptor NKp30, reveals the identity of two different soluble isoforms during normal human pregnancy. Immunobiology. (2018) 223:57–63. 10.1016/j.imbio.2017.10.012 29055565

[71] Matta J Baratin M Chiche L Forel JM Cognet C Thomas G. Induction of B7-H6, a ligand for the natural killer cell-activating receptor NKp30, in inflammatory conditions. Blood. (2013) 122:394–404. 10.1182/blood-2013-01-481705 23687088

[72] Vulpis E Cecere F Molfetta R Soriani A Fionda C Peruzzi G. Genotoxic stress modulates the release of exosomes from multiple myeloma cells capable of activating NK cell cytokine production: role of HSP70/TLR2/NF-kB axis. Oncoimmunology. (2017) 6:e1279372. 10.1080/2162402X.2017.1279372 28405503 PMC5384384

[73] Gobbo J Marcion G Cordonnier M Dias AMM Pernet N Hammann A. Restoring Anticancer immune response by targeting tumor-derived exosomes with a HSP70 peptide aptamer. J Natl Cancer Inst. (2016) 108:1–11. 10.1093/jnci/djv330 26598503

[74] Hedlund M Nagaeva O Kargl D Baranov V Mincheva-Nilsson L. Thermal- and oxidative stress causes enhanced release of NKG2D ligand-bearing immunosuppressive exosomes in leukemia/lymphoma T and B cells. PLoS ONE. (2011) 6:e16899. 10.1371/journal.pone.0016899 21364924 PMC3045385

[75] Vulpis E Soriani A Cerboni C Santoni A Zingoni A. Cancer exosomes as conveyors of stress-induced molecules: new players in the modulation of NK cell response. Int J Mol Sci. (2019) 20:E611. 10.3390/ijms20030611 30708970 PMC6387166

[76] Waldhauer I Steinle A. Proteolytic release of soluble UL16-binding protein 2 from tumor cells. Cancer Res. (2006) 66:2520–6. 10.1158/0008-5472.CAN-05-2520 16510567

[77] Waldhauer I Goehlsdorf D Gieseke F Weinschenk T Wittenbrink M Ludwig A. Tumor-associated MICA is shed by ADAM proteases. Cancer Res. (2008) 68:6368–76. 10.1158/0008-5472.CAN-07-6768 18676862

[78] Boutet P Aguera-Gonzalez S Atkinson S Pennington CJ Edwards DR Murphy G. Cutting edge: the metalloproteinase ADAM17/TNF-alpha-converting enzyme regulates proteolytic shedding of the MHC class I-related chain B protein. J Immunol. (2009) 182:49–53. 10.4049/jimmunol.182.1.49 19109134

[79] Liu G Atteridge CL Wang X Lundgren AD Wu JD. The membrane type matrix metalloproteinase MMP14 mediates constitutive shedding of MHC class I chain-related molecule A independent of A disintegrin and metalloproteinases. J Immunol. (2010) 184:3346–50. 10.4049/jimmunol.0903789 20208009 PMC3191873

[80] Chitadze G Lettau M Bhat J Wesch D Steinle A Furst D. Shedding of endogenous MHC class I-related chain molecules A and B from different human tumor entities: heterogeneous involvement of the “a disintegrin and metalloproteases” 10 and 17. Int J Cancer. (2013) 133:1557–66. 10.1002/ijc.28174 23526433

[81] Chitadze G Bhat J Lettau M Janssen O Kabelitz D. Generation of soluble NKG2D ligands: proteolytic cleavage, exosome secretion and functional implications. Scand J Immunol. (2013) 78:120–9. 10.1111/sji.12072 23679194

[82] Zoller T Wittenbrink M Hoffmeister M Steinle A. Cutting an NKG2D ligand short: cellular processing of the peculiar human NKG2D ligand ULBP4. Front Immunol. (2018) 9:620. 10.3389/fimmu.2018.00620 29651291 PMC5884875

[83] Winer A Adams S Mignatti P. Matrix metalloproteinase inhibitors in cancer therapy: turning past failures into future successes. Mol Cancer Ther. (2018) 17:1147–55. 10.1158/1535-7163.MCT-17-0646 29735645 PMC5984693

[84] Mochizuki S Okada Y. ADAMs in cancer cell proliferation and progression. Cancer Sci. (2007) 98:621–8. 10.1111/j.1349-7006.2007.00434.x 17355265 PMC11160018

[85] Siemens DR Hu N Sheikhi AK Chung E Frederiksen LJ Pross H. Hypoxia increases tumor cell shedding of MHC class I chain-related molecule: role of nitric oxide. Cancer Res. (2008) 68:4746–53. 10.1158/0008-5472.CAN-08-0054 18559521

[86] Barsoum IB Hamilton TK Li X Cotechini T Miles EA Siemens DR. Hypoxia induces escape from innate immunity in cancer cells via increased expression of ADAM10: role of nitric oxide. Cancer Res. (2011) 71:7433–41. 10.1158/0008-5472.CAN-11-2104 22006996

[87] Heo W Lee YS Son CH Yang K Park YS Bae J. Radiation-induced matrix metalloproteinases limit natural killer cell-mediated anticancer immunity in NCI-H23 lung cancer cells. Mol Med Rep. (2015) 11:1800–6. 10.3892/mmr.2014.2918 25385045

[88] Zingoni A Vulpis E Nardone I Soriani A Fionda C Cippitelli M. Targeting NKG2D and NKp30 ligands shedding to improve NK cell-based immunotherapy. Crit Rev Immunol. (2016) 36:445–60. 10.1615/CritRevImmunol.2017020166 28845754

[89] Isernhagen A Schilling D Monecke S Shah P Elsner L Walter L. The MICA-129Met/Val dimorphism affects plasma membrane expression and shedding of the NKG2D ligand MICA. Immunogenetics. (2016) 68:109–23. 10.1007/s00251-015-0884-8 26585323 PMC4728179

[90] Zingoni A Vulpis E Cecere F Amendola MG Fuerst D Saribekyan T. MICA-129 dimorphism and soluble MICA are associated with the progression of multiple myeloma. Front Immunol. (2018) 9:926. 10.3389/fimmu.2018.00926 29765374 PMC5938351

[91] Aguera-Gonzalez S Gross CC Fernandez-Messina L Ashiru O Esteso G Hang HC. Palmitoylation of MICA, a ligand for NKG2D, mediates its recruitment to membrane microdomains and promotes its shedding. Eur J Immunol. (2011) 41:3667–76. 10.1002/eji.201141645 21928280 PMC3709245

[92] Komander D Rape M. The ubiquitin code. Annu Rev Biochem. (2012) 81:203–29. 10.1146/annurev-biochem-060310-170328 22524316

[93] Bettermann K Benesch M Weis S Haybaeck J. SUMOylation in carcinogenesis. Cancer Lett. (2012) 316:113–25. 10.1016/j.canlet.2011.10.036 22138131

[94] Flotho A Melchior F. Sumoylation: a regulatory protein modification in health and disease. Annu Rev Biochem. (2013) 82:357–85. 10.1146/annurev-biochem-061909-093311 23746258

[95] Varshavsky A. The ubiquitin system, autophagy, and regulated protein degradation. Annu Rev Biochem. (2017) 86:123–28. 10.1146/annurev-biochem-061516-044859 28654326

[96] Molfetta R Gasparrini F Santoni A Paolini R. Ubiquitination and endocytosis of the high affinity receptor for IgE. Mol Immunol. (2010) 47:2427–34. 10.1016/j.molimm.2010.06.003 20638130

[97] Thomas M Boname JM Field S Nejentsev S Salio M Cerundolo V. Down-regulation of NKG2D and NKp80 ligands by Kaposi's sarcoma-associated herpesvirus K5 protects against NK cell cytotoxicity. Proc Natl Acad Sci USA. (2008) 105:1656–61. 10.1073/pnas.0707883105 18230726 PMC2234200

[98] Nice TJ Coscoy L Raulet DH. Posttranslational regulation of the NKG2D ligand Mult1 in response to cell stress. J Exp Med. (2009) 206:287–98. 10.1084/jem.20081335 19171762 PMC2646581

[99] Nice TJ Deng W Coscoy L Raulet DH. Stress-regulated targeting of the NKG2D ligand Mult1 by a membrane-associated RING-CH family E3 ligase. J Immunol. (2010) 185:5369–76. 10.4049/jimmunol.1000247 20870941 PMC3001296

[100] Fuertes MB Girart MV Molinero LL Domaica CI Rossi LE Barrio MM. Intracellular retention of the NKG2D ligand MHC class I chain-related gene A in human melanomas confers immune privilege and prevents NK cell-mediated cytotoxicity. J Immunol. (2008) 180:4606–14. 10.4049/jimmunol.180.7.4606 18354183

[101] Aguera-Gonzalez S Boutet P Reyburn HT Vales-Gomez M. Brief residence at the plasma membrane of the MHC class I-related chain B is due to clathrin-mediated cholesterol-dependent endocytosis and shedding. J Immunol. (2009) 182:4800–8. 10.4049/jimmunol.0800713 19342658

[102] Bilotta MT Abruzzese MP Molfetta R Scarno G Fionda C Zingoni A. Activation of liver X receptor up-regulates the expression of the NKG2D ligands MICA and MICB in multiple myeloma through different molecular mechanisms. FASEB J. (2019) 33:9489–504. 10.1096/fj.201900319R 31125275

[103] Fernandez-Messina L Reyburn HT Vales-Gomez M. A short half-life of ULBP1 at the cell surface due to internalization and proteosomal degradation. Immunol Cell Biol. (2016) 94:479–85. 10.1038/icb.2016.2 26732147

[104] Gong J Fang L Liu R Wang Y Xing J Chen Y. UPR decreases CD226 ligand CD155 expression and sensitivity to NK cell-mediated cytotoxicity in hepatoma cells. Eur J Immunol. (2014) 44:3758–67. 10.1002/eji.201444574 25209846

[105] Zitti B Molfetta R Fionda C Quatrini L Stabile H Lecce M. Innate immune activating ligand SUMOylation affects tumor cell recognition by NK cells. Sci Rep. (2017) 7:10445. 10.1038/s41598-017-10403-0 28874810 PMC5585267

[106] Molfetta R Milito ND Zitti B Lecce M Fionda C Cippitelli M. The Ubiquitin-proteasome pathway regulates Nectin2/CD112 expression and impairs NK cell recognition and killing. Eur J Immunol. (2019) 49:873–83. 10.1002/eji.201847848 30888046

[107] Costello RT Sivori S Marcenaro E Lafage-Pochitaloff M Mozziconacci MJ Reviron D. Defective expression and function of natural killer cell-triggering receptors in patients with acute myeloid leukemia. Blood. (2002) 99:3661–7. 10.1182/blood.V99.10.3661 11986221

[108] Garcia-Iglesias T Del Toro-Arreola A Albarran-Somoza B Del Toro-Arreola S Sanchez-Hernandez PE Ramirez-Duenas MG. Low NKp30, NKp46 and NKG2D expression and reduced cytotoxic activity on NK cells in cervical cancer and precursor lesions. BMC Cancer. (2009) 9:186. 10.1186/1471-2407-9-186 19531227 PMC2704222

[109] Charrier M Mezquita L Lueza B Dupraz L Planchard D Remon J. Circulating innate immune markers and outcomes in treatment-naive advanced non-small cell lung cancer patients. Eur J Cancer. (2019) 108:88–96. 10.1016/j.ejca.2018.12.017 30648633

[110] Carlsten M Norell H Bryceson YT Poschke I Schedvins K Ljunggren HG. Primary human tumor cells expressing CD155 impair tumor targeting by down-regulating DNAM-1 on NK cells. J Immunol. (2009) 183:4921–30. 10.4049/jimmunol.0901226 19801517

[111] Sanchez-Correa B Gayoso I Bergua JM Casado JG Morgado S Solana R. Decreased expression of DNAM-1 on NK cells from acute myeloid leukemia patients. Immunol Cell Biol. (2012) 90:109–15. 10.1038/icb.2011.15 21383766

[112] Groh V Wu J Yee C Spies T. Tumour-derived soluble MIC ligands impair expression of NKG2D and T-cell activation. Nature. (2002) 419:734–8. 10.1038/nature01112 12384702

[113] Oppenheim DE Roberts SJ Clarke SL Filler R Lewis JM Tigelaar RE. Sustained localized expression of ligand for the activating NKG2D receptor impairs natural cytotoxicity *in vivo* and reduces tumor immunosurveillance. Nat Immunol. (2005) 6:928–37. 10.1038/ni1239 16116470

[114] Molfetta R Quatrini L Capuano C Gasparrini F Zitti B Zingoni A c-Cbl regulates MICA- but not ULBP2-induced NKG2D down-modulation in human NK cells. Eur J Immunol. (2014) 44:2761–70. 10.1002/eji.201444512 24846123

[115] Molfetta R Quatrini L Zitti B Capuano C Galandrini R Santoni A. Regulation of NKG2D expression and signaling by endocytosis. Trends Immunol. (2016) 37:790–802. 10.1016/j.it.2016.08.015 27667711

[116] Quatrini L Molfetta R Zitti B Peruzzi G Fionda C Capuano C. Ubiquitin-dependent endocytosis of NKG2D-DAP10 receptor complexes activates signaling and functions in human NK cells. Sci Signal. (2015) 8:ra108. 10.1126/scisignal.aab2724 26508790

[117] Paolini R Serra A Molfetta R Piccoli M Frati L Santoni A. Tyrosine kinase-dependent ubiquitination of CD16 zeta subunit in human NK cells following receptor engagement. Eur J Immunol. (1999) 29:3179–87. 10.1002/(SICI)1521-4141(199910)29:10<3179::AID-IMMU3179>3.0.CO;2-9 10540329

[118] Capuano C Romanelli M Pighi C Cimino G Rago A Molfetta R. Anti-CD20 therapy acts via FcgammaRIIIA to diminish responsiveness of human natural killer cells. Cancer Res. (2015) 75:4097–108. 10.1158/0008-5472.CAN-15-0781 26229120

[119] Capuano C Pighi C Molfetta R Paolini R Battella S Palmieri G. Obinutuzumab-mediated high-affinity ligation of FcgammaRIIIA/CD16 primes NK cells for IFNgamma production. Oncoimmunology. (2017) 6:e1290037. 10.1080/2162402X.2017.1290037 28405525 PMC5384385

[120] Paolini R Molfetta R Piccoli M Frati L Santoni A. Ubiquitination and degradation of Syk and ZAP-70 protein tyrosine kinases in human NK cells upon CD16 engagement. Proc Natl Acad Sci USA. (2001) 98:9611–6. 10.1073/pnas.161298098 11493682 PMC55500

[121] Borrego F Lopez-Beltran A Pena J Solana R. Downregulation of Fc gamma receptor IIIA alpha (CD16-II) on natural killer cells induced by anti-CD16 mAb is independent of protein tyrosine kinases and protein kinase C. Cell Immunol. (1994) 158:208–17. 10.1006/cimm.1994.1268 8087866

[122] Grzywacz B Kataria N Verneris MR. CD56(dim)CD16(+) NK cells downregulate CD16 following target cell induced activation of matrix metalloproteinases. Leukemia. (2007) 21:356–9. 10.1038/sj.leu.2404499 17251901

[123] Romee R Foley B Lenvik T Wang Y Zhang B Ankarlo D. NK cell CD16 surface expression and function is regulated by a disintegrin and metalloprotease-17 (ADAM17). Blood. (2013) 121:3599–608. 10.1182/blood-2012-04-425397 23487023 PMC3643761

[124] Peruzzi G Femnou L Gil-Krzewska A Borrego F Weck J Krzewski K. Membrane-type 6 matrix metalloproteinase regulates the activation-induced downmodulation of CD16 in human primary NK cells. J Immunol. (2013) 191:1883–94. 10.4049/jimmunol.1300313 23851692 PMC3745217

[125] Zhou Q Gil-Krzewska A Peruzzi G Borrego F. Matrix metalloproteinases inhibition promotes the polyfunctionality of human natural killer cells in therapeutic antibody-based anti-tumour immunotherapy. Clin Exp Immunol. (2013) 173:131–9. 10.1111/cei.12095 23607800 PMC3694543

[126] Waldhauer I Steinle A. NK cells and cancer immunosurveillance. Oncogene. (2008) 27:5932–43. 10.1038/onc.2008.267 18836474

[127] Zingoni A Cecere F Vulpis E Fionda C Molfetta R Soriani A. Genotoxic stress induces senescence-associated ADAM10-dependent release of NKG2D MIC ligands in multiple myeloma cells. J Immunol. (2015) 195:736–48. 10.4049/jimmunol.1402643 26071561

[128] Arai J Goto K Tanoue Y Ito S Muroyama R Matsubara Y. Enzymatic inhibition of MICA sheddase ADAM17 by lomofungin in hepatocellular carcinoma cells. Int J Cancer. (2018) 143:2575–83. 10.1002/ijc.31615 29873070

[129] Mullooly M McGowan PM Crown J Duffy MJ. The ADAMs family of proteases as targets for the treatment of cancer. Cancer Biol Ther. (2016) 17:870–80. 10.1080/15384047.2016.1177684 27115328 PMC5004698

[130] Camodeca C Nuti E Tepshi L Boero S Tuccinardi T Stura EA. Discovery of a new selective inhibitor of A Disintegrin And Metalloprotease 10 (ADAM-10) able to reduce the shedding of NKG2D ligands in Hodgkin's lymphoma cell models. Eur J Med Chem. (2016) 111:193–201. 10.1016/j.ejmech.2016.01.053 26871660

[131] Wolpert F Tritschler I Steinle A Weller M Eisele G. A disintegrin and metalloproteinases 10 and 17 modulate the immunogenicity of glioblastoma-initiating cells. Neuro Oncol. (2014) 16:382–91. 10.1093/neuonc/not232 24327582 PMC3922520

[132] Huang B Sikorski R Sampath P Thorne SH. Modulation of NKG2D-ligand cell surface expression enhances immune cell therapy of cancer. J Immunother. (2011) 34:289–96. 10.1097/CJI.0b013e31820e1b0d 21389869 PMC3073622

[133] Kloess S Huenecke S Piechulek D Esser R Koch J Brehm C. IL-2-activated haploidentical NK cells restore NKG2D-mediated NK-cell cytotoxicity in neuroblastoma patients by scavenging of plasma MICA. Eur J Immunol. (2010) 40:3255–67. 10.1002/eji.201040568 21061445

[134] Ferrari de Andrade L Tay RE Pan D Luoma AM Ito Y Badrinath S. Antibody-mediated inhibition of MICA and MICB shedding promotes NK cell-driven tumor immunity. Science. (2018) 359:1537–42. 10.1126/science.aao0505 29599246 PMC6626532

[135] Lombana TN Matsumoto ML Berkley AM Toy E Cook R Gan Y. High-resolution glycosylation site-engineering method identifies MICA epitope critical for shedding inhibition activity of anti-MICA antibodies. mAbs. (2019) 11:75–93. 10.1080/19420862.2018.1532767 30307368 PMC6343778

[136] Mishra HK Pore N Michelotti EF Walcheck B. Anti-ADAM17 monoclonal antibody MEDI3622 increases IFNgamma production by human NK cells in the presence of antibody-bound tumor cells. Cancer Immunol Immunother. (2018) 67:1407–16. 10.1007/s00262-018-2193-1 29978334 PMC6126979

[137] Chen D Frezza M Schmitt S Kanwar J Dou QP. Bortezomib as the first proteasome inhibitor anticancer drug: current status and future perspectives. Curr Cancer Drug Targets. (2011) 11:239–53. 10.2174/156800911794519752 21247388 PMC3306611

[138] Kisselev AF van der Linden WA Overkleeft HS. Proteasome inhibitors: an expanding army attacking a unique target. Chem Biol. (2012) 19:99–115. 10.1016/j.chembiol.2012.01.003 22284358 PMC3503453

[139] Vandross A. Proteasome inhibitor-based therapy for treatment of newly diagnosed multiple myeloma. Semin Oncol. (2017) 44:381–84. 10.1053/j.seminoncol.2018.01.002 29935899

[140] Morrow JK Lin HK Sun SC Zhang S. Targeting ubiquitination for cancer therapies. Future Med Chem. (2015) 7:2333–50. 10.4155/fmc.15.148 26630263 PMC4976843

[141] Niu C Jin H Li M Zhu S Zhou L Jin F. Low-dose bortezomib increases the expression of NKG2D and DNAM-1 ligands and enhances induced NK and gammadelta T cell-mediated lysis in multiple myeloma. Oncotarget. (2017) 8:5954–64. 10.18632/oncotarget.13979 27992381 PMC5351604

[142] Soriani A Zingoni A Cerboni C Iannitto ML Ricciardi MR Di Gialleonardo V. ATM-ATR-dependent up-regulation of DNAM-1 and NKG2D ligands on multiple myeloma cells by therapeutic agents results in enhanced NK-cell susceptibility and is associated with a senescent phenotype. Blood. (2009) 113:3503–11. 10.1182/blood-2008-08-173914 19098271

[143] Fionda C Stabile H Molfetta R Soriani A Bernardini G Zingoni A. Translating the anti-myeloma activity of Natural Killer cells into clinical application. Cancer Treat Rev. (2018) 70:255–64. 10.1016/j.ctrv.2018.10.005 30326421

[144] Bernstock JD Ye D Gessler FA Lee YJ Peruzzotti-Jametti L Baumgarten P. Topotecan is a potent inhibitor of SUMOylation in glioblastoma multiforme and alters both cellular replication and metabolic programming. Sci Rep. (2017) 7:7425. 10.1038/s41598-017-07631-9 28785061 PMC5547153

[145] Fukuda I Ito A Hirai G Nishimura S Kawasaki H Saitoh H. Ginkgolic acid inhibits protein SUMOylation by blocking formation of the E1-SUMO intermediate. Chem Biol. (2009) 16:133–40. 10.1016/j.chembiol.2009.01.009 19246003

[146] Suzawa M Miranda DA Ramos KA Ang KK Faivre EJ Wilson CG. A gene-expression screen identifies a non-toxic sumoylation inhibitor that mimics SUMO-less human LRH-1 in liver. eLife. (2015) 4:e09003. 10.7554/eLife.09003 26653140 PMC4749390

[147] Yang Y Xia Z Wang X Zhao X Sheng Z Ye Y. Small-molecule inhibitors targeting protein SUMOylation as novel anticancer compounds. Mol Pharmacol. (2018) 94:885–94. 10.1124/mol.118.112300 29784649

